# On Decorating a Honeycomb AlN Monolayer with Hydrogen and Fluorine Atoms: Ab Initio and Experimental Aspects

**DOI:** 10.3390/ma17030616

**Published:** 2024-01-27

**Authors:** Edward Ferraz de Almeida, Anelia Kakanakova-Georgieva, Gueorgui Kostov Gueorguiev

**Affiliations:** 1 Center for Exact Sciences and Technologies, Federal University of West of Bahia, Rua Bertioga, 892, Morada Nobre I, Barreiras 47810-059, Brazil; edward.almeida@ufob.edu.br; 2 Department of Physics, Chemistry and Biology (IFM), Linköping University, SE-581 83 Linköping, Sweden; anelia.kakanakova@liu.se

**Keywords:** group IIIA nitrides, 2D AlN, functionalization, hydrogenation, fluorination

## Abstract

Mono- and few-layer hexagonal AlN (h-AlN) has emerged as an alternative “beyond graphene” and “beyond h-BN” 2D material, especially in the context of its verification in ultra-high vacuum Scanning Tunneling Microscopy and Molecular-beam Epitaxy (MBE) experiments. However, graphitic-like AlN has only been recently obtained using a scalable and semiconductor-technology-related synthesis techniques, such as metal–organic chemical vapor deposition (MOCVD), which involves a hydrogen-rich environment. Motivated by these recent experimental findings, in the present work, we carried out ab initio calculations to investigate the hydrogenation of h-AlN monolayers in a variety of functionalization configurations. We also investigated the fluorination of h-AlN monolayers in different decoration configurations. We find that a remarkable span of bandgap variation in h-AlN, from metallic properties to nar-row-bandgap semiconductor, and to wide-bandgap semiconductor can be achieved by its hy-drogenation and fluorination. Exciting application prospects may also arise from the findings that H and F decoration of h-AlN can render some such configurations magnetic. We complemented this modelling picture by disclosing a viable experimental strategy for the fluorination of h-AlN.

## 1. Introduction

Mono- and few-layer hexagonal AlN (h-AlN) has emerged as an alternative “beyond graphene” and “beyond h-BN” 2D material, especially in the context of its properties that were verified through Ultra-High Vacuum Scanning Tunneling Microscopy (STM) [1] and Molecular-Beam Epitaxy (MBE) [2] experiments. These experiments have demonstrated that ultrathin films of h-AlN can be built up from 12 monolayers [1] or alternatively, from 5 to 6 monolayers [2], on substrates such as Ag(111) and Si(111). Ultrathin films of h-AlN (as well as h-GaN, h-BeO, h-ZnO, h-ZnS, and h-SiC) were first predicted by theoretical calculations and indicated as graphitic nanofilm precursors for the crystal growth of wurtzite materials [3]. Predictive first-principles calculations establish the structure and dynamic stability of mono- and multilayer structures of h-AlN [4,5,6,7]. Their modeling further extends to surface functionalization of h-AlN with *hydrogen* (*H*) [8,9,10] and *fluorine* (*F*) [11,12,13] atoms as a strategy to tune its respective structural and electronic properties. Of particular note for future device applications are the predictions that fluorinated h-AlN may exhibit enhanced piezoelectricity and semi-metallicity [12]. More recently, co-decoration of h-AlN monolayers with F atoms on one side and H atoms on the other side, i.e., a Janus-type chemical functionalization, has been discussed in comparison with fully hydrogenated h-AlN monolayers [14].

Previously, the effect of hydrogenation and fluorination in the modification of the structural and electronic properties of graphene has been well documented in the context of theoretical and experimental studies [15]. Fully hydrogenated graphene (called graphane) and fully fluorinated graphene (called fluorographene) show sp^3^ bond hybridization rather than the sp^2^ bond hybridization in graphene [15,16,17]. Most importantly, these sp^3^-bonded graphene derivatives allow for the opening of the band gap of graphene and altering its electrical conductivity and magnetism [15,16,17,18]. Systematic first-principles calculations have been performed to investigate the modification of the structural, electronic, and magnetic properties of h-BN through hydrogenation and fluorination, finding notable differences compared to graphene, as summarized in [19]. Semi-fluorinated monolayers of h-BN and h-ZnO have been predicted to become ferromagnetic and robust half-metals, with the potential to create building blocks for semiconductor-based spintronics [20]. More recently, the effect of hydrogenation and fluorination of h-BN monolayers has been investigated in terms of their carrier mobility [21].

h-BN, as well as graphene, are naturally occurring layered materials, giving access to monolayers by, e.g., mechanical exfoliation [22], and therefore enables experimental studies on chemical functionalization, among others [23]. h-AlN is not a naturally occurring layered material. Few-layer h-AlN ultrathin layers have initially been stabilized under the stringent conditions of ultra-high vacuum STM [1] and MBE [2] experiments. Graphitic-like AlN has recently been obtained using scalable and semiconductor-technology-related synthesis techniques such as metal–organic chemical vapor deposition (MOCVD) [24]. For the first time, and by MOCVD, a graphitic-like GaN monolayer in a buckled geometry was also reported [25]. As a typical feature, these MOCVD processes were carried out in a hydrogen-rich environment.

Motivated by these recent experimental findings, in the present work, we investigated the hydrogenation of h-AlN monolayers in a variety of decoration configurations. We focus on their structural properties, including buckling, which can be used as a fingerprint of h-AlN in scanning electron microscopic studies, as well as their electronic and magnetic properties, which are important from the point of view of band engineering and application. We also investigated the fluorination of h-AlN monolayers in different decoration configurations. As is well known, fluorination has significant advantages over hydrogenation due to the high electronegativity of the *fluorine* atoms, which has enabled the efficient opening of the band gap of graphene [26]. We present a preliminary study of original experiments on the fluorination of AlN thin films, which extends the scope of our work on the decoration of an AlN honeycomb monolayer with *hydrogen* and *fluorine* atoms.

## 2. Computational Methodology

The framework adopted for the present calculations was density functional theory (DFT) within its generalized gradient approximation (GGA) with exchange–correlation energy and spin polarization, considering a collinear calculation, to evaluate the magnetization along the z axis. The level of theory adopted was Perdew–Burke–Ernzerhof (PBE) as implemented in the Quantum Espresso code [27,28]. The calculation set-up employed Plane-Wave basis sets and projector-augmented wave method (PAW) pseudo-potentials [29,30] for the aluminum, nitrogen, *hydrogen*, and *fluorine* atoms.

Initially, an h-AlN monolayer was optimized. Its unit cell consisted of an aluminum atom positioned at (0, 0, 0) and a nitrogen atom at (1/3, 2/3, 0). While the hexagonal symmetry group of the 3D wurtzite AlN is P6m2, the h-AlN and its decorated structures are characterized by lower 2D space groups: C_6_v for the flat pristine h-AlN monolayer and C_2V_ for the decorated buckled h-AlN monolayers. An equilibrium network parameter for the unit cell of 3.1513 Å was found. As typically done in simulations of monolayer type structures, the value of the parameter c was chosen to be equal to 15 Å [16], thus avoiding self-interactions along the z axis. A suitable supercell (2 × 2 × 1) was adopted to study different functional configurations. The geometrically optimized h-AlN monolayer (exhibiting an Al–N bond length of 1.82 Å) was subsequently used to assemble all model systems of decorated h-AlN monolayers considered in this work. These model systems fall into three classes:

(i) Fully hydrogenated h-AlN monolayers (H-AlN-H), with H atoms bonded to Al sites on one side of the monolayer as well as to N atoms on the other side.
(ii) Semi-decorated h-AlN monolayers with H atoms bonded to Al sites (H-AlN) and alternatively to N sites (H-NAl). This class of model systems also includes a semi-decorated h-AlN monolayer with *fluorine* atoms bonded to Al sites (F-AlN). The alternative semi-decorated h-AlN monolayer with F atoms bonded to N sites is usually disregarded [12] because of the orbital repulsion between the F and N atoms due to their higher electronegativity. We, however, included the corresponding weakly bound F-NAl complex in the present work.
(iii) Janus-type decorated h-AlN monolayers with F atoms bonded to Al sites on one side of the monolayer and H atoms bonded to N sites on the other side (F-AlN-H).

In each of these three classes, model systems corresponding to all permutations of H and F atoms and their bonding sites on the h-AlN monolayer were investigated.

The adsorption energy ($E_{ads}$) of decorating atoms, as used in the discussions, is defined as follows:

(1) $$E_{ads}\left[X-AlN-Y\right]={\{E}_{tot}\left[X-AlN-Y\right]-E_{tot}\left[h-AlN\right]-n_{X}\mu_{X}-n_{Y}\mu_{Y}\}/(n_{X}+n_{Y})$$

whereby *E_tot_* is the the total energy per supercell of the model system including the bonded/adsorbed H and/or F atoms; *n_X_* and *n_Y_* correspond to thenumber of types of *X* and *Y* atoms per supercell; and *μ_X_* and *μ_Y_* are the chemical potentials of type *X* and *Y* atoms (*X*, *Y* = H, F). 

## 3. Results and Discussion

The presentation of the calculation results begins with a presentation of the structural and electronic properties of the pristine, non-decorated h-AlN monolayer. These characteristics are included in the present discussion for the purposes of benchmarking and reliability testing of the chosen level of theory.

### 3.1. Pristine h-AlN Monolayer

**Figure 1:**
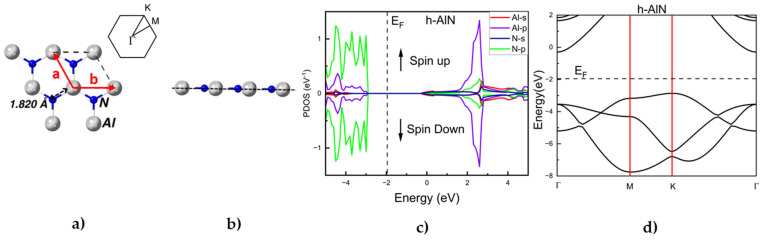
Optimized structure of h-AlN monolayer: (**a**) top view and (**b**) side view. The dashed line indicates the typical planarity of this structure. In panel (**a**), the 2D Bravais lattice unit vectors a = b have an angle between the vectors of 120° and are shown in red. The inset in panel (**a**) depicts the primitive unit cell as well as the high symmetry points of the Brillouin zone (BZ) with the path along which the band structure was evaluated; (**c**) projected density of states (PDOS; eV^−1^) as function of energy (eV); (**d**) band structure (the dashed line represents the Fermi level (E_F_)).

As displayed in Figure 1a,b, the pristine h-AlN monolayer exhibits a hexagonal (honeycomb) symmetry, planar (flat) geometry with an Al-N bond length of 1.820 Å, and a lattice parameter of 3.152 Å. These values agree with previously reported findings [4,5,6,7].

Figure 1c shows the projected density of states (PDOS) for a pristine h-AlN monolayer. It is well known that the p-orbitals of the N atoms are a major contributor to the region of band energies −4 eV and −2 eV while the p-orbitals of Al atoms strongly contribute to the region around −4 eV [5]. The top of the valence band is dominated by the N lone pairs while in the regions above the Fermi level, the role of the p-orbitals of Al atoms is most significant. The h-AlN monolayer reveals an sp^2^ hybridization defined by the unoccupied *pz* orbitals of the Al atoms and the N lone pairs. The partial charges of the Al atoms and the N atoms are 1.9172|*e*| and 5.9942|*e*|, quantitatively describing the ionic character of the Al-N bond. The PDOS (Figure 1c) illustrates the symmetry between the spin-up and spin-down states. This result implies an absence of a magnetic moment, confirming that the pristine h-AlN is a non-magnetic material. In Figure 1d, the band structure of a pristine h-AlN monolayer is shown. The calculated band gap of 2.60 eV (Table 1) is indirect and agrees with previous results for h-AlN [7,10,14].

### 3.2. Fully Hydrogenated H-AlN-H Monolayer

**Figure 2:**
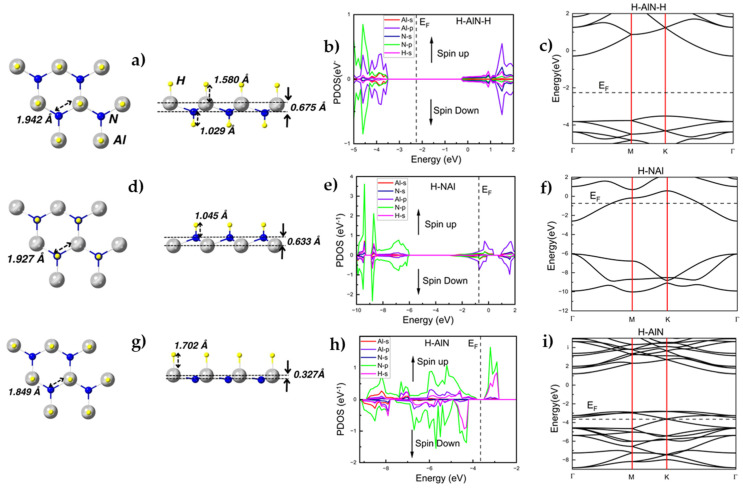
(**a**) Top and side views of optimized structures of fully hydrogenated H-AlN-H. The characteristic bond lengths and the corresponding buckling parameters are also indicated. (**b**) PDOS (eV^−1^) as function of energy (eV) and (**c**) band structure (the dashed line represents the Fermi level (E_F_)) of fully hydrogenated H-AlN-H. (**d**–**f**) Optimized structure, PDOS, and band structure of semi-hydrogenated H-NAl. (**g**–**i**) Optimized structure, PDOS, and band structure of semi-hydrogenated H-AlN.

The structure of a fully hydrogenated H-AlN-H monolayer is shown in Figure 2a. The full hydrogenation of h-AlN stretches its Al-N bond length from 1.820 Å to 1.942 Å (Figure 2a, Table 1). The length of the N-H bonds is 1.029 Å (Table 1), which is a value comparable to the N-H bond length in ammonia (1.01 Å); the H-Al bond length of 1.580 Å (Table 1) is corroborated by previous results, e.g., by Wang et al. [14].

The hydrogenation of h-AlN leads to buckling which is strongest (0.675 Å, Table 1) in the case of the fully hydrogenated H-AlN-H monolayer (Figure 2a) and significantly weaker for the H-AlN monolayer (0.327 Å, Figure 2c, Table 1), which can be attributed to the weaker Al-H bonds. Remarkably, the Al-N-H bond angle in the case of the fully hydrogenated H-AlN-H monolayer is 110.397°—a value suggesting a departure from the pure sp^2^ hybridization and toward an sp^3^-type hybridization.

The partial density of states (PDOS) and the band structure of the fully hydrogenated H-AlN-H monolayer are shown in Figure 2b and in Figure 2c, respectively. The symmetry observed for the spin-up and spin-down states (Figure 2b) suggests that, similar to the case of the pristine h-AlN, the fully hydrogenated H-AlN-H monolayer is a non-magnetic 2D material.

Another PDOS feature (Figure 2b) of H-AlN-H is due to the s orbital of the H atoms which, together with the p orbitals of the Al atoms, strongly contribute to the energy level of the valence band maximum (VBM). The full hydrogenation of the h-AlN monolayer attains states in the Fermi level region, which leads to an increase in the bandgap to 3.231 eV (Table 1), i.e., an increase of about 24% when compared to the band gap of h-AlN. The indirect type of bandgap is preserved using hydrogenation. The significant increase of ~1/4 of the bandgap accompanying the full hydrogenation coverage of the h-AlN bandgap is a result which qualitatively matches the bandgap modification resulting from the hydrogenation of other group IIIA nitride monolayers such as h-BN [19]. The calculated adsorption energy of a single H atom to h-AlN averages −0.28 eV/atom (Table 1) depending on the adsorption site, thus confirming the process of H-decoration of h-AlN as an exothermic chemical reaction.

### 3.3. Semi-Hydrogenated H-NAl and H-AlN Monolayers

We first considered the semi-hydrogenated H-NAl model system, where the H atoms are bonded to all N atoms of an h-AlN monolayer (Figure 2d). The H-N bond length is 1.045 Å (Table 1), while this type of semi-hydrogenation slightly lengthens the Al-N bond length to 1.927 Å (Table 1). The buckling parameter of the H-NAl monolayer is 0.633 Å (Figure 2d, Table 1), a value not much different from the buckling of the fully hydrogenated H-AlN-H model system. Significantly, the Al-N-H bond angle of 109.176° indicates a transition from sp^2^ to sp^3^ hybridization while the mixed covalent–ionic character of bonding is preserved.

The PDOS (Figure 2e) and the band structure (Figure 2f) of the semi-hydrogenated H-NAl model system reveal that the p orbitals of the Al and the N atoms, as well as the s orbital of the H atoms, dominate the energy levels in proximity to the Fermi level, thus decidedly pointing to the metallic character of H-NAl. Also noteworthy is that the averaged adsorption energy of a decorating H atom in the H-NAl model systems equals 0.41 eV/atom; this positive value is indicative for an endothermic reaction (i.e., needs an energy investment) during the H-NAl formation process.

As documented in the literature, it is the semi-decorated group IIIA nitride systems that may exhibit magnetic properties [31]. Thus, to verify whether each of the semi-decorated systems studied in our work is indeed magnetic, we analyzed all three configurations: the non-magnetic (NM), the ferromagnetic (FM), and the antiferromagnetic (AFM) configurations.

As illustrated by the asymmetric behavior of the PDOS (Figure 2e) and resulting from the dislocation of the p-orbitals of the Al atoms toward the Fermi level, the magnetic moment inherent to the H-NAl monolayer is equal to −0.50$\mu_{B},$ which is consistent with the FM configuration (as depicted in Figure 2d). The FM and AFM H-NAl configurations only differ in stability by 2.45 meV/atom. Their co-existence, or the advantage of one over the other, may be defined by thermal, kinetic, and substrate-related factors during the synthesis of the material.

An alternative semi-hydrogenated configuration, whereby the H atoms are bonded to the Al atoms of an h-AlN monolayer, can also be formed (Figure 2g). The corresponding H-Al bond length is equal to 1.702 Å (Figure 2g, Table 1), while the Al-N bonds lengthen to 1.849 Å (Table 1, an insignificant change in comparison to the Al-N bond of 1.82 Å that characterizes a pristine h-AlN monolayer). Furthermore, the optimized H-AlN reveals a buckling parameter of 0.327 Å (Figure 2g, Table 1) which is just about the half of the buckling parameter of 0.675 Å inherent to the fully hydrogenated H-AlN-H. Our findings regarding the degree of buckling of H-AlN are corroborated by the results obtained by Y. Ma et al. [31], where a level of theory and an implementation differing from the ones employed in the present work were used. The reduced buckling of H-AlN in comparison to H-NAl and to H-AlN-H indicates a retention of a stronger covalent character of bonding (Al-N-H bond angle of 100.173° and N-Al-N angle of 116.949°) in H-AlN than in H-NAl and H-AlN-H.

The PDOS and band structure of H-AlN are presented in Figure 2h and Figure 2i, respectively. Notably, H-AlN is characterized by domination of the region in proximity to the Fermi level by the p orbital of the N atoms together with the s orbital of the H atoms, thus revealing the metallic character of H-AlN. The H adsorption energy averages 0.34 eV/atom (Table 1), suggesting an endothermic formation reaction which is predicted to be more favorable in terms of necessary energy investment to synthesize H-AlN than is the case for H-NAl (with average adsorption energy of 0.41 eV/atom, Table 1).

Figure 2h shows the decidedly asymmetric behavior of the spin-up/spin-down states for H-AlN resulting in a magnetic moment equal to −4.00$\mu_{B}$, which is consistent with the FM configuration (as depicted in Figure 2g) which is more stable by 18.77 meV/atom than the H-AlN AFM configuration. Thus, H-AlN appears to be ferromagnetic (FM), with its N p and H s orbitals contributing most strongly to its magnetization.

### 3.4. Fluorination of h-AlN

**Figure 3:**
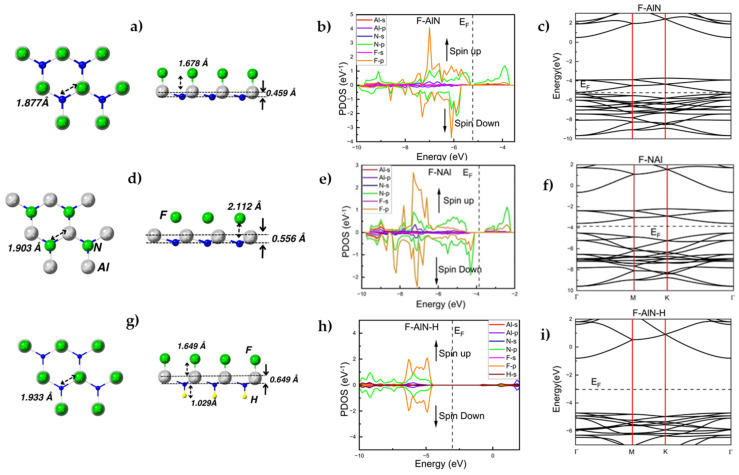
(**a**) Top and side views of optimized structure of semi-fluorinated F-AlN. The characteristic bond lengths and the corresponding buckling parameters are also indicated. (**b**) PDOS (eV^−1^) as function of energy (eV) and (**c**) its band structure (the dashed line represents the Fermi level (E_F_)) of semi-fluorinated F-AlN. (**d**–**f**) Optimized structure, PDOS, and band structure of weakly bonded semi-hydrogenated F:NAl. (**g**–**i**) Optimized structure, PDOS, and band structure of Janus-type F-AlN-H.

The F-AlN monolayer where the F atom decoration results in formation of Al-F bonds with a bond length of 1.678 Å is shown in Figure 3a. As a result of the fluorination and the F-Al bond formation, the monolayer’s Al-N bond undergoes insignificant stretching to 1.877 Å (Figure 3a, Table 1; compared to the Al-N bond length of 1.82 Å in h-AlN), while the F-AlN system acquires a buckling of 0.459 Å (Figure 3a, Table 1).

As illustrated in Figure 3b (PDOS) and in Figure 3c (band structure), electronic structure changes are also observed, the most important of which being a strong narrowing of the bandgap to the value of 0.164 eV (Table 1) thus making F-AlN a narrow-gap semiconductor. Due to the strongly asymmetric behavior of the PDOS of F-AlN (Figure 3b), its magnetic moment is equal to −4.00$\mu_{B}$ consistent with the FM configuration (as depicted in Figure 3a). The FM and AFM F-AlN configurations differ in stability by 6.03 meV/atom. Their co-existence, or the advantage of one over the other, may be defined by thermal, kinetic, and substrate-related factors during the synthesis of the material. The adsorption energy of the F atoms in the case of F-AlN is equal to −0.93 eV/atom (Table 1), which is an indication for a strong exothermic process and thus for a significant energetic advantage for its experimental realization.

The F atom decoration at the N sites of the h-AlN monolayer is illustrated in Figure 3d. The orbital repulsion between the F and N atoms due to their high electronegativities leads to an equilibrium F:N distance of 2.112 Å without forming any well-defined F-N chemical bond. Yet, the F:N orbital interaction forms a weakly bound F:h-NAl complex (F:h-NAl being, due to its bonding, a more appropriate designation of such a system than F-NAl), whose formation leads to characteristic structural, electronic, and magnetic changes in comparison to a pristine h-AlN monolayer. The structural consequences include a stretching of its Al-N bond length to a value of 1.903 Å (Table 1) as well as introducing buckling of the F:h-NAl complex, described by a parameter equal to 0.556 Å (Table 1). As illustrated in Figure 3e (PDOS) and in Figure 3f (band structure), electronic structure changes in the F:h-NAl complex do occur. Governed by the p orbitals of the N and F atoms, the resulting modification of the band structure in proximity to the Fermi level reduces the width of band gap to 0.921 eV (Table 1) but also changes the bandgap type from an indirect to a direct one. The adsorption energy of the F atoms of −0.60 eV/atom (Table 1) points to an exothermic reaction for the formation of the weakly bound complex of F:h-NAl. This can be attributed to the extreme chemical reactivity and electronegativity of F. The calculated magnetic moment is equals to 4.00$\mu_{B}$, which is consistent with the FM configuration (as depicted in Figure 3d). The FM and AFM F-AlN configurations differ in stability by 9.30 meV/atom. Their co-existence, or the advantage of one over the other, may be defined by thermal, kinetic, and substrate-related factors during the synthesis of the material. It is worth noting that the F decoration of group IIIA nitride monolayers was recently investigated in the case of a honeycomb BN monolayer, h-BN, [32] whereby the formation of any F-N bonds was conditioned to the previous F decoration of the boron atoms which makes the N sites in the h-BN network less electronegative and thus benefits F-N bond formation. In the case of h-AlN, due to Al being a more typical metal than B, i.e., Al is less electronegative than B (electronegativity of 1.61 for Al vs. 2.04 for B), Al-N bond polarization and the corresponding charge distribution within the h-AlN monolayer concedes less local electronegativity to the N sites thus favoring the formation of the weakly bound complex of F:h-NAl even without any decoration at the Al sites.

The optimized h-AlN monolayer with Janus-type decoration by H and F (F-AlN-H) is shown in Figure 3g. It is fully described in terms of structure by its F-Al bond length of 1.649 Å, its H-N bond length of 1.029 Å, its Al-N bond length of 1.933 Å, and its buckling parameter of 0.649 Å (Figure 3g, Table 1). The F-Al and H-N bond lengths are indicative of strong bonding of the decoration atoms to the AlN monolayer.

Figure 3h (PDOS) and Figure 3i (band structure) illustrate that the p orbitals of the F and N atoms are dominant in proximity to the Fermi level. The bandgap turns to a direct bandgap with a width of 3.914 eV, a value which clearly lists the Janus-type F-AlN-H monolayer among the wide-bandgap semiconductors. F-AlN-H is predicted to be non-magnetic (Figure 3h). This Janus-type monolayer is also remarkable by exhibiting an averaged (between all F and H decorating atoms) adsorption energy of −1.35 eV/atom (Table 1), the highest adsorption energy value among all h-AlN monolayers decorated with H and F studied in the present work.

As already noted in the introduction, graphene and h-BN have been the subject of intense research over the past two decades and their structural and electronic properties as well as a variety of processing methods developed for their synthesis, doping, functionalization, and combination with other materials to form heterostructures have been widely reviewed [17,22]. In comparison, h-AlN is predominantly studied in the field of predictive first-principles calculations. While theoretical studies reveal the importance of hydrogenation and fluorination to alter the structural, electronic, and magnetic properties of graphene, h-BN, and h-AlN (including the results of this study), experimental strategies on how to achieve chemical functionalization, e.g., fluorination of h-BN [32], are much rarer. Regarding graphene, we indicate a method for graphene fluorination by irradiating fluoropolymer-covered graphene with a laser [26]. In this method, the laser-induced decomposition of the fluoropolymer is believed to produce highly active intermediates such as CF_x_ and F radicals, which can react with sp^2^-hybridized graphene and form a C-F sp^3^ bond [26]. In this reported example, graphene was prepared by chemical vapor deposition (CVD) on a Cu foil and transferred to a SiO_2_/Si substrate for post-processing and characterization. We emphasize the CVD approach for the synthesis of graphene and other 2D materials such as h-BN and h-AlN due to its compatibility with the fabrication technologies and device fabrication processes for semiconductor materials. In this regard, we note the developments that led to the successful 3D synthesis of AlN on SiC and graphitized SiC substrates [33,34] and 2D synthesis [24] in confinement at a graphene/SiC interface based on MOCVD in our experiments. We thus identified and set to seek a potentially practical approach to AlN fluorination that is technologically motivated [35]. The fluorination of AlN was carried out in a system that has been favorably applied to the synthesis of *fluorine*-containing amorphous carbon films in an Ar/tetrafluoromethane (CF4)-containing medium [36,37]. Chemically reactive CFn radicals are expected to result from the dissociation of CF4 on the heated AlN surface. The presence of *fluorine* atoms on the AlN surface was confirmed by SIMS [35]. We expect this approach to be a viable strategy for the fluorination of h-AlN as well, given that h-AlN has already been obtained as an intercalated layer at a graphene/SiC interface [24] and was air-stable and, in principle, accessible for further characterization and chemical/heat treatment.

In summary, we present the picture of decorating an h-AlN monolayer with *hydrogen* and *fluorine* atoms; how this leads to significant structural modification whereas buckling may constitute a useful feature for the experimental identification of decorated structures; and how it creates opportunities for h-AlN bandgap engineering. These opportunities reveal a remarkable span from metallic properties (as are the cases of both the H-AlN and H-NAl semi-hydrogenated configurations) to narrow-bandgap semiconductor (F-AlN) and to wide-bandgap semiconductor properties (H-AlN-H, and F-AlN-H). Exciting application prospects arise from the findings that H and F decoration of h-AlN can render some configurations (H-AlN, H-NAl, F-AlN) magnetic. We complement this modelling picture by conferring a viable experimental strategy for the hydrogenation and fluorination of h-AlN obtained by semiconductor-technology-relevant MOCVD.

## Figures and Tables

**Table 1:** Calculated structural, electronic, and energy characteristics of all studied model systems of an h-AlN monolayer decorated with H and F atoms and including a pristine, non-decorated h-AlN monolayer. Al-N stands for the bond length in Å. In the case of H and F atom decoration, H/F-Al and N-H denote the corresponding bond lengths in Å. ∆ is the buckling parameter. Bandgap E_g_ and adsorption energy E_ad_ are given in (eV) and (eV/atom), respectively. μ is the magnetic moment in $\mu_{B}$. The non-zero μ values in the table indicate those decorated systems that exhibit ferromagnetic (FM) properties. The systems found to be non-magnetic are described by μ = 0.

System	Al-N	H/F-Al	N-H	∆	E_g_	E_ad_	μ
h-AlN	1.820	-	-	-	2.600	-	0
H-AlN-H	1.942	1.580	1.029	0.675	3.231	−0.28	0
H-NAl	1.927	-	1.045	0.633	0	0.41	−0.50
H-AlN	1.849	-	1.702	0.327	0	0.34	−4.00
F-AlN	1.877	1.678	-	0.459	0.164	−0.93	−4.00
F:NAl	1.903	-	(2.112)	0.556	0.921	−0.60	−4.00
F-AlN-H	1.933	1.649	1.029	0.649	3.914	−1.35	0

## Data Availability

All data relevant to this study are available in the paper. Any additional details related to calculation details are available upon reasonable request to the authors.
